# Comparing perceived psychosocial working conditions of nurses and physicians in two university hospitals in Germany with other German professionals - feasibility of scale conversion between two versions of the German Copenhagen Psychosocial Questionnaire (COPSOQ)

**DOI:** 10.1186/s12995-020-00277-w

**Published:** 2020-08-20

**Authors:** Anke Wagner, Matthias Nübling, Antje Hammer, Tanja Manser, Monika A. Rieger, on behalf of the WorkSafeMed Consortium, E. Luntz, M. A. Rieger, H. Sturm, A. Wagner, A. Hammer, T. Manser, P. Martus, M. Holderried

**Affiliations:** 1 Institute of Occupational and Social Medicine and Health Services Research, University Hospital of Tübingen, Wilhelmstraße 27, 72074 Tübingen, Germany; 2 Freiburg Research Centre for Occupational Sciences (FFAW GmbH), Bertoldstr. 63, 79098 Freiburg, Germany; 3 Institute of Patient Safety, University Hospital of Bonn, Venusberg-Campus 1, 53127 Bonn, Germany; 4 FHNW School of Applied Psychology, University of Applied Sciences and Arts Northwestern Switzerland, Riggenbachstrasse 16, 4600 Olten, Switzerland

**Keywords:** Psychosocial working conditions, Hospitals, Copenhagen psychosocial questionnaire, Physicians, Nurses, Explorative statistical analysis, COPSOQ database, Reference data

## Abstract

**Background:**

In 2015, the WorkSafeMed study assessed, amongst others, perceived psychosocial working conditions in nurses (*n* = 567) and physicians (*n* = 381) from two German university hospitals using scales from the German standard version of the COPSOQ (Copenhagen Psychosocial Questionnaire). This standard version is based on the international COPSOQ I and II. Since 2017, a further developed version of the German COPSOQ (G-COPSOQ III) has been available and data from this version are stored in the German COPSOQ database. The aim of the present study was to compare scales depicting perceived psychosocial workloads and strain in hospital staff from the WorkSafeMed study with reference data (hospital care nurses, general hospital physicians, reference values across all occupations) from the German COPSOQ database (2012–2017). As preliminary work, we explored whether a conversion of COPSOQ scales based on data from the WorkSafeMed study to the G-COPSOQ III scales was possible.

**Methods:**

We applied a multistep approach for conversion. First, we compared 17 COPSOQ scales used in the WorkSafeMed study with the corresponding scales from the G-COPSOQ III according to content and then decided if a conversion was appropriate. If possible, we converted WorkSafeMed scales - the converted scales comprised the same content and number of items as in G-COPSOQ III. An explorative statistical analysis for each original and converted WorkSafeMed scale followed detecting possible statistical and relevant differences between the scales. We then compared converted WorkSafeMed scales with reference data from the German COPSOQ database.

**Results:**

Based on the comparison undertaken according to content, a conversion was possible for 16 scales. Using the data from the WorkSafeMed study, the statistical analysis showed only differences between original and converted COPSOQ scales *“control over working time”* (mean 40.2 vs. 51.8, d_Cohen_ = 0.56) and *“social relations”* (mean 55.6 vs. 41.8, d_Cohen_ = − 0.55). Comparing converted WorkSafeMed scales with reference data revealed higher values for *“quantitative demands”*, *“work-privacy-conflict”,* and *“job satisfaction”* in the WorkSafeMed sample*.*

**Conclusions:**

The conversion of WorkSafeMed scales was appropriate, allowed a comparison with three reference values in the German COPSOQ database and revealed some implications for improving psychosocial working conditions of nurses and physicians in university hospitals in Germany.

## Background

The Copenhagen Psychosocial Questionnaire (COPSOQ) is a well-known and widely accepted instrument for measuring psychosocial working conditions in different professional branches. COPSOQ I was originally developed in Denmark in 1997, capturing a broad range of psychosocial working conditions [1]. According to the authors, the questionnaire should fulfil the following criteria: “…theory-based, but not attached to one specific theory…, …consist of dimensions related to different levels of analysis (organization, department, job, person-work interface, and individual), …include dimensions related to work tasks, the organization of work, interpersonal relations, cooperation and leadership, …cover potential work stressors, as well as resources such as support, feedback, commitment, and good health, …should be comprehensive…, …should be generic, meaning that it should be applicable in all sectors of the labor market..., the medium-length and short versions should be “user friendly” with regard to work environment professionals and respondents (employees)” [1]. In 2004/2005, a validation study took place in Denmark to develop the second version of the Copenhagen Psychosocial Questionnaire (COPSOQ II) [2]. Since 2013, an international study has been carried out by researchers of the COPSOQ network (www.copsoq-network.org) to develop the third version of the Copenhagen Psychosocial Questionnaire (COPSOQ III) [3]. This study contained in total 23.361 data records and more than 10.000 data records from Germany. The authors demonstrated within their study on the basis of the core items a comparable reliability of COPSOQ II and COPSOQ III. The developed COPSOQ III questionnaire contains new occupational health topics and intends to ensure international comparability [3]. Meanwhile, there are several validation studies on COPSOQ II and COPSOQ III from different countries that report satisfying values for reliability and validity [3–8].

In Germany, a first standard version of the COPSOQ questionnaire based on COPSOQ I was established and tested in 2004 in a sample of 2561 employees [4]. As part of the validation study, a shortened version of the instrument was developed to have a suitable instrument for assessing psychosocial working conditions [9, 10]. This shortened version (2005) included 87 items and 25 aspects and has found widespread use as a paper and pencil questionnaire and as an online tool [10]. In 2011, new occupational health topics from the international COPSOQ II were included into the questionnaire, such as social capital, trust, and justice. Until 2017, the German standard version based on COPSOQ I and II was continuously further developed and completed. In 2017, the new German standard version based on COPSOQ III was made available. This German standard version based on COPSOQ III comprises 85 items and 26 aspects, and the psychometric validation of the questionnaire has recently been carried out [11]. To ease understanding, the following abbreviations for the different versions of the German COPSOQ standard version will be used throughout the rest of the article: G-COPSOQ I is the German standard version after the validation study based on COPSOQ I. G-COPSOQ II is the second German standard version based on COPSOQ I and II; G-COPSOQ III is the German standard version based on COPSOQ III.

In most cases, G-COPSOQ III utilizes the same items as in G-COPSOQ II, meaning there are only small differences in content between the scales used in both versions. There are mainly differences regarding the number of items. Table 1 shows an overview of the scales and number of items used in both versions.

**Table 1:** Scales and number of items used in G-COPSOQ II and G-COPSOQ III

Scales	G-COPSOQ II	G-COPSOQ III
	N items	N items
Domain: Demands		
Quantitative demands	4 items	3 items
Emotional demands	3 items	2 items
Demands for hiding emotions	2 items	2 items
Work-privacy-conflict	5 items	2 items
Domain: Influence and development		
Influence at work	4 items	3 items
Degree of freedom at work / control over working time	4 items	2 items
Possibilities for development	4 items	3 items
Meaning of work	3 items	2 items
Workplace commitment	4 items	2 items
Domain: Interpersonal relations and leadership		
Predictability	2 items	2 items
Role clarity	4 items	3 items
Role conflicts	4 items	3 items
Quality of leadership	4 items	4 items
Social support	4 items	4 items
Feedback	2 items	2 items
Social relations	2 items	Single item
Sense of community	3 items	2 items
Bullying	Single item	Single item
Trust & Justice	4 items	4 items
Further parameters		
Insecurity over employment	4 items	4 items
Domain: Strain (effects, outcomes)		
Intention to leave	Single item	Single item
Job satisfaction	7 items	6 items
General health	Single item	Single item
Burnout (CBI)	6 items	3 items
Overcommitment	3 items	Single item

In addition to the continuous development of the German COPSOQ questionnaire, new data were added to a steadily growing German COPSOQ database to enable the development of job-specific profiles of psychosocial factors at work [10]. Thus, institutions can compare their results with results from other jobs and with their job-specific reference values in the COPSOQ database [10]. In 2020, the COPSOQ database contains more than 400.000 reference values from various occupational groups (e.g. manufacturers, technicians, teachers, social workers, waste management…) [10, 12]. As staff surveys using the COPSOQ are voluntary for companies and institutions, the COPSOQ database is not representative. This means that for some professions there is a high number of reference values, while other professions are not well represented. On top it has to be borne in mind, that in Germany staff surveys using the COPSOQ are often performed within the so-called psychosocial risk assessment i.e. as an occupational health and safety activity [13] and not within a study. For the hospital sector there exist currently a sufficient number of reference values for both nurses (> than 8000 cases) and physicians (> than 2000 cases) to perform comparisons. Yet, the values are not classified according to the type or size of hospital (e.g. university hospital, general hospital).

Since 2017, the previous comparative dataset from surveys with G-COPSOQ I and II was transformed to the content of G-COPSOQ III, and only information fitting to or data assessed with this version (G-COPSOQ III) are now stored in the German COPSOQ database. Therefore, only scales and single items based on this version can be compared in the current COPSOQ database, whereas comparative data for studies using scales from G-COPSOQ II are no longer available. In general, it is important for further studies to find a way for the comparisons of results gathered with different versions of a questionnaire. One study compare COPSOQ I and COPSOQ II regarding the influence of psychosocial factors on a specific health outcome (need for recovery) [14]. But there are currently no studies that compare and convert scales from different versions of the COPSOQ questionnaire. Since the COPSOQ questionnaire is commonly used in Germany in both, science and occupational health and safety activities, and enables the continued comparison of results from studies that used the G-COPSOQ II questionnaire with data from the COPSOQ database, to us the question arose whether scales from G-COPSOQ II can be converted to scales from G-COPSOQ III.

## Methods

### Aim of the study

Our interest in this methodological question originates from the WorkSafeMed study as we wanted to compare scales depicting perceived psychosocial workloads and strain in hospital staff from this study in two university hospitals performed in 2015 with reference data (hospital care nurses, general hospital physicians as well as the reference value across all occupations) from the German COPSOQ database (2012–2017). The comparison with reference data can be used to derive some implications for improving psychosocial working conditions for nurses and physicians in university hospitals in Germany.

As the WorkSafeMed study used the G-COPSOQ II questionnaire and not the G-COPSOQ III questionnaire, we applied a multistep approach for conversion between these two versions to finally enable a comparison between the converted scales from the WorkSafeMed study and reference data (hospital care nurses, general hospital physicians as well as the reference value across all occupations) from the German COPSOQ database.

### Design and setting

The WorkSafeMed study (“***Work****ing conditions,* ***safe****ty culture and patient safety in hospitals – what predicts the safety of the* ***med****ication process”)* was a cross-sectional, multicenter, mixed-methods project conducted between 2014 and 2017 [15–18]. The study included a staff survey using a standardized paper-based questionnaire to assess psychosocial working conditions (G-COPSOQ II), patient and occupational safety cultures [15, 16], a chart review to evaluate the quality of the medication process [17] and the explorative correlation analysis of questionnaire and routine data to depict workload and quality of care [18].

### Data collection, response rates, and sample characteristics

We conducted the survey of nursing staff and physicians at two German university hospitals between April 2015 and July 2015. All inpatient units (except for intensive care and psychiatric units) which treat at least 500 patients per year were included [16]. The paper-based questionnaire was distributed to a total of 2512 physicians and nurses. After about 2 to 4 weeks, one written reminder was sent and, if necessary, one oral reminder was communicated [16]. In the WorkSafeMed study, a total of 995 questionnaires were returned [16]. The overall response rate was 39.6% [16]. In total, we collected data from 37 departments, including 73 units. The sample consisted of 381 physicians and 567 nurses [16]. Forty-seven persons participated who either belonged to another professional group (19 persons) or gave no information on their professional status (28 persons) [16]. Table 2 describes the sample of nurses and physicians in the WorkSafeMed study. In the sample of nurses, more females and persons without supervisor functions were represented than in the physicians’ sample. The mean age of the participating nurses was 38.6 years (±11.9) and the average work experience was around 16.5 years (±11.7). In the physician sample, there were slightly more men than women. The physicians’ mean age was 36.1 years (±8.2). Compared to nurses, physicians had less work experience of about 9.0 years (±7.8).

**Table 2:** Description of the sample in the WorkSafeMed study (*N* = 995)

Profession	Variable	Categories	n (%)
Nurses *n* = 567	Gender	female male missing values	470 (82.9%) 87 (15.3%) 10 (1.8%)
	Supervisor function	yes no missing values	71 (12.5%) 491 (86.6%) 5 (0.9%)
	Direct patient contact	yes no	565 (99.6%) 2 (0.4%)
	Age (in years)	≤30 31–49 ≥50	193 (34.0%) 197 (34.7%) 177 (31.2%)
	Work experience (in years)	0–10 11–20 ≥21	224 (39.5%) 135 (23.8%) 208 (36.7%)
	Work experience in the hospital (in years)	0–10 11–20 ≥21 missing values	250 (44.1%) 115 (20.3%) 201 (35.4%) 1 (0.2%)
Physicians *n* = 381	Gender	female male missing values	167 (43.8%) 202 (53.0%) 12 (3.1%)
	Supervisor function	yes no missing values	123 (32.3%) 247 (64.8%) 11 (2.9%)
	Direct patient contact	yes no missing value	377 (99.0%) 3 (0.8%) 1 (0.3%)
	Age (in years)	≤30 31–49 ≥50	109 (28.6%) 214 (56.2%) 58 (15.2%)
	Work experience (in years)	0–10 11–20 ≥21	253 (66.4%) 80 (21.0%) 48 (12.6%)
	Work experience in the hospital (in years)	0–10 11–20 ≥21	261 (68.5%) 41 (10.8%) 79 (20.7%)

### Questionnaire

**Fig. 1:**
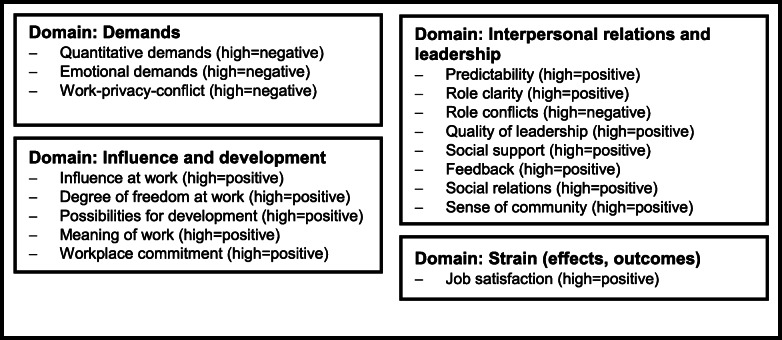
Content of the G-COPSOQ II scales in the WorkSafeMed study

The paper-based questionnaire for the staff survey in the WorkSafeMed study used common and validated instruments [15, 16]. To assess *psychosocial working conditions*, we employed 17 scales of the G-COPSOQ II [9, 19]. Items were answered on a 4-point or 5-point Likert scale. Reverse coding was necessary for one item (“Do you work separate from your colleagues?”) before scale calculation. To calculate scores, we followed the recommendation for COPSOQ transformation [10] and answering scales were transformed into scores ranging from 0 (minimum value, “do not agree at all”) to 100 points (maximum value, “fully agree”). Depending on the wording of items within each COPSOQ scale, maximum values can be positive (high = positive) or negative (high = negative). An overview of the scales used in our questionnaire is shown in Fig. 1.

### Ethics and confidentially issues

Ethics approval was received from the ethical committees at the two university hospitals involved (Reference numbers #350/14 and #547/2014BO1). During the survey, participants were asked for informed consent. Participants were also informed that the study was voluntary and that they could withdraw their consent at any time. Only anonymized data were used for the statistical analysis [16].

### Data analysis

Before data analysis, missing values were imputed with NORM 2.03 software using the Expectation-Maximization-algorithm [20, 21]. Items of the COPSOQ scales were placed into two separate imputation groups (group 1: psychosocial working conditions/group 2: leadership) [16]. Respondents with missing values of > 30% of items were excluded prior to the imputation because of the limited data quality. We excluded responses with missing values in imputation group 1 (psychological working conditions) *n* = 4 (0,4%), and in imputation group 2 (leadership): *n* = 42 (4,2%) [16].

### Preliminary work

We applied, as preliminary work for the comparison with reference data, a multistep approach to convert original WorkSafeMed scales as formulated in the G-COPSOQ II to the scales from the current German COPSOQ database (G-COPSOQ III).

- *Comparison at a level of content*

In a first step*,* we compared 17 G-COPSOQ II scales used in the WorkSafeMed study with the 17 corresponding scales from the G-COPSOQ III at a content level (including single items and response categories). The results of the comparison between the two versions (G-COPSOQ II versus G-COPSOQ III) can be summarized as follows:

- **Domain Demands**: In this domain, we found some differences at the item level for two scales (*“quantitative demands”* and *“work-privacy-conflict”*). For example, the number of items for both scales was reduced in G-COPSOQ III and the response categories for the scale *“work-privacy-conflict”* were modified. We discovered large differences for one scale (*“emotional demands”*). For this scale, two previous items were no longer used. Instead, a completely new item was introduced.
- **Domain Influence and development**: In this domain, we found some differences at the item level for all five scales. The number of items for all five scales was reduced. A slightly different item formulation was used in two scales (*“influence at work”* and *“workplace commitment”*), and response categories were modified for one scale (*“possibilities for development”*). Additionally, the scale name *“degree of freedom at work”* was renamed to *“control over working time”*.
- **Domain Interpersonal relations and leadership**: We also discovered some differences in this domain. The number of items for four scales (*“role clarity”*, *“role conflicts”*, *“social relations”*, and *“sense of community”*) was reduced and there was an added response category for two scales (*“social relations”* and *“sense of community”*). For four scales (*“predictability”*, *“social support”*, *“feedback”*, and *“quality of leadership”*), only minimal differences were found, and the number of items remained the same. We found a slightly different item formulation and an added response category for three scales (*“social support”*, *“feedback”*, and *“quality of leadership”*).
- **Domain Strain**: There were some differences at the item level for the scale *“job satisfaction”*. The number of items was reduced, a slightly different item formulation was used, and we found modified response categories.

Table 3 shows the comparison of scales, single items, and response categories of the two versions.

**Table 3:** Comparison of scales, single items and response categories - G-COPSOQ II versus G-COPSOQ III

G-COPSOQ II		G-COPSOQ III		Summary of amendments
Scales and items	Response categories	Scales and items	Response categories	
**Domain: Demands**				
Quantitative demands (4 items) − Do you have to work very fast? − Is your workload unevenly distributed so it piles up? − How often do you not have time to complete all your work tasks? − Do you have to do overtime?	always / often / sometimes / seldom / never, hardly ever	Quantitative demands (3 items) − Do you have to work very fast? − How often do you not have time to complete all your work tasks? − Do you have to do overtime?	always / often / sometimes / seldom / never, hardly ever	− reduced from 4 to 3 items
Emotional demands (3 items) − Does your work put you in emotionally disturbing situations? − Do you get emotionally involved in your work? − Is your work emotionally demanding?	always / often / sometimes / seldom / never, hardly ever	Emotional demands (2 items) − Do you have to deal with other people’s personal problems as part of your work?	always / often / sometimes / seldom / never, hardly ever	− introduction of a new item − modified response categories
		− Is your work emotionally demanding?	to a very large extent / to a large extent / some - what / to a small extent / to a very small extent	
Work-privacy-conflict (5 items) − The demands of my work interfere with my home and family life. − The amount of time my job takes up makes it difficult to fulfil my family responsibilities. − Things I want to do at home do not get done because of the demands my job puts on me. − My job produces strain that makes it difficult to fulfill family duties. − Due to work-related duties, I have to make changes to my plans for family activities.	strongly agree / slightly agree / neither agree nor disagree / slightly disagree / strongly disagree	Work-privacy-conflict (2 items) − The demands of my work interfere with my home and family life. − The amount of time my job takes up makes it difficult to fulfil my family responsibilities.	to a very large extent / to a large extent / some - what / to a small extent / to a very small extent	− reduced from 5 to 2 items − modified response categories
**Domain: Influence and development**				
Influence at work (4 items) − Do you have a large degree of influence concerning your work? − Do you have a say in choosing who you work with? − Can you influence the amount of work assigned to you? − Do you have any influence on what you do at work?	always / often / sometimes / seldom / never, hardly ever	Influence at work (3 items) − Do you have a large degree of influence on the decisions concerning your work? − Can you influence the amount of work assigned to you? − Do you have any influence on what you do at work?	always / often / sometimes / seldom / never, hardly ever	− reduced from 4 to 3 items − slightly different formulation for one item
Degree of freedom at work (4 items) − Can you decide when to take a break? − Can you take holidays more or less when you wish? − Can you leave your work to have a chat with a colleague? − If you have some private business, is it possible for you to leave your place of work for half an hour without special permission?	always / often / sometimes / seldom / never, hardly ever	Control over working time (2 items) − Can you decide when to take a break? − Can you take holidays more or less when you wish?	always / often / sometimes / seldom / never, hardly ever	− reduced from 4 to 2 item − new scale designation (control over working time)
Possibilities for development (4 items) − Is your work varied? − Does your work require you to take the initiative? − Do you have the possibility of learning new things through your work? − Can you use your skills or expertise in your work?	always / often / sometimes / seldom / never, hardly ever	Possibilities for development (3 items) − Is your work varied?	always / often / sometimes / seldom / never, hardly ever	− reduced from 4 to 3 items − for 2 items modified response categories
		− Do you have the possibility of learning new things through your work? − Can you use your skills or expertise in your work?	to a very large extent / to a large extent / some - what / to a small extent / to a very small extent	
Meaning of work (3 items) − Is your work meaningful? − Do you feel that the work you do is important? − Do you feel motivated and involved in your work?	to a very large extent / to a large extent / some - what / to a small extent / to a very small extent	Meaning of work (2 items) − Is your work meaningful? − Do you feel that the work you do is important?	to a very large extent / to a large extent / some - what / to a small extent / to a very small extent	− reduced from 3 to 2 items
Workplace commitment (4 items) − Are you proud to be part of this organization? − Do you enjoy telling others about your place of work? − Do you feel that the problems at your place of work are yours too? − Do you feel that your place of work is of great personal importance to you?	to a very large extent / to a large extent / some - what / to a small extent / to a very small extent	Workplace commitment (2 items) − Are you proud of being part of this company? − Do you enjoy telling others about your place of work?	to a very large extent / to a large extent / some - what / to a small extent / to a very small extent	− reduced from 4 to 2 items − slightly different formulation for one item
**Domain: Interpersonal relations and leadership**				
Predictability (2 items) − At your place of work, are you informed well in advance concerning for example important decisions, changes, or plans for the future? − Do you receive all the information you need in order to do your work well?	to a very large extent / to a large extent / some - what / to a small extent / to a very small extent	Predictability (2 items) − At your place of work, are you informed well in advance concerning for example important decisions, changes, or plans for the future? − Do you receive all the information you need in order to do your work well?	to a very large extent / to a large extent / some - what / to a small extent / to a very small extent	− same number of items − slightly different item formulation
Role clarity (4 items) − Do you know exactly how much say you have at work? − Does your work have clear objectives? − Do you know exactly which areas are your responsibility? − Do you know exactly what is expected of you at work?	to a very large extent / to a large extent / some - what / to a small extent / to a very small extent	Role clarity (3 items) − Does your work have clear objectives? − Do you know exactly which areas are your responsibility? − Do you know exactly what is expected of you at work?	to a very large extent / to a large extent / some - what / to a small extent / to a very small extent	− reduced from 4 to 3 items
Role conflicts (4 items) − Do you do things at work, which are accepted by some people but not by others? − Are contradictory demands placed on you at work? − Do you sometimes have to do things, which ought to have been done in a different way? − Do you sometimes have to do things, which seem to you to be unnecessary?	to a very large extent / to a large extent / some - what / to a small extent / to a very small extent	Role conflicts (3 items) − Are contradictory demands placed on you at work? − Do you sometimes have to do things, which ought to have been done in a different way? − Do you sometimes have to do things, which seem to you to be unnecessary?	to a very large extent / to a large extent / some - what / to a small extent / to a very small extent	− reduced from 4 to 3 items
Quality of leadership (4 items) To what extent would you say that your immediate superior… − ...makes sure that the individual member of staff has good development opportunities? − ...gives high priority to job satisfaction? − …is good at work planning? − …is good at solving conflicts?	to a very large extent / to a large extent / some - what / to a small extent / to a very small extent	Quality of leadership (4 items) To what extent would you say that your immediate superior… − ...makes sure that the members of staff have good development opportunities? − ...gives high priority to job satisfaction? − …is good at work planning? − …is good at solving conflicts?	to a very large extent / to a large extent / some - what / to a small extent / to a very small extent / I don’t have a superior	− same number of items − slightly different item formulation − new response category (I don’t have a superior)
Social support (4 items) − How often do you get help and support from your colleagues? − How often are your colleagues willing to listen to your problems at work? − How often do you get help and support from your nearest superior? − How often is your immediate superior willing to listen to your work-related problems?	always / often / sometimes / seldom / never, hardly ever	Social support (4 items) − How often do you get help and support from your colleagues, if needed? − How often are your colleagues willing to listen to your problems at work, if needed? − How often do you get help and support from your immediate superior, if needed? − How often is your immediate superior willing to listen to your problems at work, if needed?	always / often / sometimes / seldom / never, hardly ever / I don’t have a superior, colleagues	− same number of items − slightly different item formulation − new response category (I don’t have a superior, colleagues)
Feedback (2 items) − How often do you talk with your superior about how well you carry out your work? − How often do you talk with your colleagues about how well you carry out your work?	always / often / sometimes / seldom / never, hardly ever	Feedback (2 items) − How often does your immediate superior talk with you about how well you carry out your work? − How often do your colleagues talk with you about how well you carry out your work?	always / often / sometimes / seldom / never, hardly ever / I don’t have a superior, colleagues	− same number of items − slightly different item formulation − new response category (I don’t have a superior, colleagues)
Social relations (2 items) − Do you work separate from your colleagues? − Is it possible for you to talk to your colleagues while you are working?	always / often / sometimes / seldom / never, hardly ever	Social relations (single item) − Is it possible for you to talk to your colleagues while you are working?	always / often / sometimes / seldom / never, hardly ever / I don’t have a superior, colleagues	− reduced from 2 to 1 item − new response category (I don’t have a superior, colleagues)
Sense of community (3 items) − Is there a good atmosphere between you and your colleagues? − Is there good co-operation between your colleagues at work? − Do you feel part of a community at your place of work?	always / often / sometimes / seldom / never, hardly ever	Sense of community (2 items) − Is there a good atmosphere between you and your colleagues? − Is there good co-operation between the colleagues at work?	always / often / sometimes / seldom / never, hardly ever / I don’t have a superior, colleagues	− reduced from 3 to 2 items − new response category (I don’t have a superior, colleagues)
**Domain: Strain (effects, outcomes)**				
Job satisfaction (7 items) Regarding your work in general. How pleased are you with… − ...your work prospects? − …the people you work with? − ...the physical working conditions? − ...the way your department is run? − ...the way your abilities are used? − ...the interest and skills involved in your job? − ...your job as a whole, everything taken into consideration?	very satisfied / satisfied / unsatisfied / highly unsatisfied	Job satisfaction (6 items) Regarding your work in general. How pleased are you with… − ...your work prospects? − …the people you work with? − ...the physical working conditions? − ...the way your group is run? − ...the way your abilities are used? − ...your job as a whole, everything taken into consideration?	very satisfied / satisfied / neither, nor / unsatisfied / highly unsatisfied	− reduced from 7 to 6 items − slightly different formulation for one item − modified response categories

After the comparison, all differences found regarding the content were discussed by the team (AW, MN and MAR) and a consensus was reached as to whether a conversion of the original scales from the WorkSafeMed dataset would be appropriate or not, i.e. the post-hoc reconstruction of the G-COPSOQ III using data assessed by the G-COPSOQ II. We decided not to convert the scale *“emotional demands”*, since the discovered differences in content were considered too comprehensive.

b) *Post-hoc reconstruction of WorkSafeMed scales*

In a second step, 16 original WorkSafeMed scales (G-COPSOQ II) were converted in accordance to the scales from the current G-COPSOQ III. For the post-hoc-reconstruction of original WorkSafeMed scales, we converted the items of a scale, which are also used for the respective scale of G-COPSOQ III, so that the scales comprised the same content and the same number of items of this version. In most cases, the same response options were used for the different versions of the questionnaire. In two cases, the response options in the G-COPSOQ III questionnaire were slightly modified. However, the differences were very marginal, so that they were not considered when converting the scales.

c) *Reliability analysis and statistical tests for assessment of differences: original WorkSafeMed scales versus converted WorkSafeMed scales*

In a third step, we conducted a reliability analysis and assessed Cronbach’s alpha for each original and newly converted WorkSafeMed scale. We thereby considered values between .70 and .90 as good [22, 23]. We then performed an explorative statistical analysis to determine whether there were statistically significant and relevant differences between the original and the newly converted WorkSafeMed scales. For detecting statistical differences, a t-test was calculated to compare the mean of the original WorkSafeMed scales with the mean of the converted WorkSafeMed scales. For the evaluation of the t-tests, an adjustment for multiple testing was applied computing Bonferroni corrected *p*-values [24] and therefore indicating *p* <  0.001 (two-sided) as statistically significant. In addition to the t-test, the effect size (d_Cohen_) was calculated to evaluate the magnitude of differences. We categorized the effect size according to Cohen’s suggestions: <.30 = small effect/difference, <.50 = medium effect/difference and ≥ .50 = large effect/ difference [25]. In accordance with previous COPSOQ studies [26, 27], we applied the following nomenclature for identifying differences in the scales: a difference of at least 5 points in the mean values of groups is considered a clear difference; a deviation of 10 or more points is considered a very clear deviation [10]. This principle is based on the effect size measure (Cohen’s d): COPSOQ scales usually have standard deviations of 15–25 points, thus 5 points represent a small to intermediate effect size of 0.2–0.33 and 10 points represent middle to strong effect sizes 0.4 to 0.66 [26].

### Comparison of converted WorkSafeMed scales with reference data

To compare results on psychosocial working conditions of the converted WorkSafeMed scales with reference data from the COPSOQ database (hospital care nurses, general hospital physicians as well as the reference value across all occupations), we performed an analysis of variance (ANOVA). For the interpretation of differences, we employed the previously described nomenclature and considered 5 points as a clear difference. Due to the high number of tests (16 scales, 2 study groups, 3 reference values), a *p*-value < 0.01 (two-sided) was established as statistically significant. In addition, we calculated the effect size (d_Cohen_) for all significant results.

All statistical analyses were performed using IBM Statistics SPSS for Windows, version 25 (IBM Corp., Armonk, NY, USA). Based on the results of the comparison undertaken with reference data, we derived some implications for improved psychosocial working conditions for nurses and physicians in university hospitals.

## Results

### Reliability analysis and statistical tests for assessment of differences: original WorkSafeMed scales versus converted WorkSafeMed scales

Based on the dataset derived from the WorkSafeMed study, the original WorkSafeMed scales and the converted WorkSafeMed scales were compared. Table 4 shows the descriptive statistics and the results of the reliability analysis, the t-test including the results for the Bonferroni correction and the effect size, as well as the applied nomenclature.

**Table 4:** Comparison of original WorkSafeMed scales and converted WorkSafeMed scales: Descriptive statistics, results of the student’s t test, effect size and, nomenclature

Scales (*n*=)	Original WorkSafeMed scales Mean (SD) Cronbach’s α	Converted WorkSafeMed scales Mean (SD) Cronbach’s α	(df) t-value^1^	Effect size d_Cohen_	Nomenclature^2^
**Domain: Demands**					
Quantitative demands (*n* = 991)	68.6 (14.0) α = .71	70.5 (14.3) α = .66	(990) 3.241	0.13	< 5 points
Work-privacy-conflict (*n* = 991)	63.8 (25.2) α = .92	63.0 (27.7) α = .88	(990) -1.116	− 0.03	< 5 points
**Domain: Influence and development**					
Influence at work (*n* = 991)	37.3 (18.8) α = .75	42.1 (19.6) α = .70	(990) 8.179***	0.25	< 5 points
Control over working time (*n* = 991)	40.2 (18.5) α = .65	51.8 (22.5) α = .41	(990) 16.528***	0.56	**> 10 points**
Possibilities for development (*n* = 991)	74.8 (15.9) α = .77	74.5 (17.2) α = .75	(990) -0.940	−0.02	< 5 points
Meaning of work (*n* = 991)	79.7 (16.8) α = .79	84.1 (17.0) α = .81	(990) 7.639***	0.26	< 5 points
Workplace commitment (*n* = 991)	53.7 (20.1) α = .75	55.2 (25.2) α = .81	(990) 1.561	0.07	< 5 points
**Domain: Interpersonal relations and leadership**					
Predictability (*n* = 991)	53.0 (17.8) α = .62	53.0 (17.8) α = .62	(990) 0.026	0.00	< 5 points
Role clarity (*n* = 991)	73.1 (15.6) α = .84	73.6 (16.0) α = .80	(990) 1.223	0.03	< 5 points
Role conflicts (*n* = 991)	48.4 (18.0) α = .73	49.2 (19.5) α = .74	(990) 1.979	0.04	< 5 points
Quality of leadership (*n* = 953)	52.0 (22.9) α = .90	52.0 (22.9) α = .90	(952) -0.053	0.00	< 5 points
Social support (*n* = 991)	65.8 (17.0) α = .76	65.8 (17.0) α = .76	(990) -0.444	0.00	< 5 points
Feedback (*n* = 991)	41.5 (21.3) α = .67	41.5 (21.3) α = .67	(990) -0.666	0.00	< 5 points
Social relations (*n* = 991)	55.6 (20,6) α = .35	41.8 (28.7) n/a (single item)	(990) 20.778***	−0.55	**> 10 points**
Sense of community (*n* = 991)	77.3 (15.0) α = .80	77.5 (14.6) α = .83	(990) 0.993	0.01	< 5 points
**Domain: Strain (effects, outcomes)**					
Job satisfaction (*n* = 991)	69.9 (11.4) α = .80	69.1 (11.9) α = .77	(990) -2.340	−0.07	< 5 points

^1***^Bonferroni-corrected *p*-values (*p* <  0.001)

^2^Nomenclature**:** differences of more than 5 points are considered relevant and presented in bold

n/a = not applicable

The reliability analysis showed similar and satisfying values of Cronbach’s α above .70 for most of the original and the converted scales. Three original WorkSafeMed scales (*“control over working time”, “predictability”, “feedback”*) and three converted WorkSafeMed scales (*“quantitative demands”, “predictability”, “feedback”*) achieved only values between .60 and .70. The original WorkSafeMed scale *“social relations”* revealed a Cronbach’s alpha of .35. Since the converted WorkSafeMed scale *“social relations”* consisted of a single item, no calculation of the Cronbach’s alpha was possible for this scale. The converted WorkSafeMed scale *“control over working time”* resulted in a Cronbach’s alpha of only .41.

The t-test revealed significant differences between original and converted WorkSafeMed scales (*p* < 0.001 after Bonferroni correction) for the following four scales: *“influence at work”*, *“control over working time”, “meaning of work”,* and *“social relations”*. The differences for *“influence at work”* (d = 0.25) and *“meaning of work”* (d = 0.26) represented small effects, while the differences for *“control over working time”* (d = 0.56) and *“social relations”* (d = − 0.55) showed a large effect. The interpretation of the nomenclature resulted in a value greater than 10 for the scales *“control over working time”* and *“social relations”,* indicating that there is a very clear difference between the original and the converted WorkSafeMed scales.

### Comparison of converted WorkSafeMed scales with reference data (German COPSOQ database)

Table 5 presents the differences in the means for all converted WorkSafeMed scales for nurses and physicians and the job-specific reference values for general hospital care nurses (COPSOQ nurses) and general hospital physicians (COPSOQ physicians), as well as the reference value across all occupations (COPSOQ all occupations).

**Table 5:** Study results for WorkSafeMed nurses and WorkSafeMed physicians and COPSOQ database reference values (COPSOQ nurses, COPSOQ physicians, COPSOQ all occupations). Scale means, standard deviations, *p*-values, and effect sizes (d_Cohen_)

Scales	WorkSafeMed nurses (converted scales)	COPSOQ nurses (COPSOQ database)	WorkSafeMed nurses vs. COPSOQ nurses		WorkSafeMed physicians (converted scales)	COPSOQ physicians (COPSOQ database)	WorkSafeMed physicians vs. COPSOQ physicians		COPSOQ all occupations (COPSOQ database)	WorkSafeMed nurses vs. all occupations		WorkSafeMed physicians vs. all occupations	
**Domain: Demands**													
	**Mean (SD)**	**Mean (SD)**	**p**	**d**	**Mean (SD)**	**Mean (SD)**	**p**	**d**	**Mean (SD)**	**p**	**d**	**p**	**d**
Quantitative demands (high = negative)	68.4 (13.9) (*n* = 564)	61.9 (15.9) (*n* = 8973)	+	−0.41	73.9 (13.9) (*n* = 380)	70.1 (16.9) (*n* = 2356)	sig.	−0.23	56.3 (19.3) (*n* = 194,073)	+	−0.63	+	−0.92
Work-privacy-conflict (high = negative)	59.8 (26.8) (*n* = 564)	55.4 (28.1) (*n* = 8969)	sig.	−0.16	68.8 (27.7) (*n* = 380)	67.7 (27.9) (*n* = 2354)			42.7 (30.5) (*n* = 194,079)	+	−0.56	+	−0.86
**Domain: Influence and development**													
Influence at work (high = positive)	41.5 (18.6) (*n* = 564)	37.3 (20.1) (*n* = 8.960	sig.	−0.21	42.7 (20.9) (*n* = 380)	42.8 (20.3) (*n* = 2354)			42.4 (22.9) (*n* = 192,670)				
Control over working time (high = positive)	51.0 (22.8) (*n* = 564)	50.5 (22.5) (*n* = 8982)			52.6 (22.0) (*n* = 380)	51.6 (22.3) (*n* = 2354)			61.5 (25.2) (*n* = 186,554)	–	0.42	–	0.35
Possibilities for development (high = positive)	70.7 (17.2) (*n* = 564)	65.9 (18.2) (*n* = 8976)	sig.	−0.27	80.1 (14.7) (*n* = 380)	75.0 (16.1) (*n* = 2359)	+	−0.32	61.9 (22.2) (*n* = 194,064)	+	−0.40	+	−0.82
Meaning of work (high = positive)	83.0 (17.2) (*n* = 564)	81.0 (18.5) (*n* = 8976)			85.9 (16.4) (*n* = 380)	81.8 (17.8) (*n* = 2359)	sig.	−0.23	74.6 (21.4) (*n* = 194,220)	+	−0.39	+	−0.53
Workplace commitment (high = positive)	49.8 (23.9) (*n* = 564)	52.7 (24.6) (*n* = 8970)			63.0 (24.6) (*n* = 380)	53.6 (23.8) (*n* = 2359)	+	−0.39	58.3 (25.4) (*n* = 193,423)	–	0.34	sig.	−0.18
**Domain: Interpersonal relations and leadership**													
Predictability (high = positive)	53.3 (16.4) (*n* = 564)	50.4 (20.1) (*n* = 8944)	sig.	−0.15	52.5 (19.3) (*n* = 380)	49.9 (20.3) (*n* = 2350)			51.3 (22.2) (*n* = 192,212)				
Role clarity (high = positive)	74.2 (15,0) (*n* = 564)	72.7 (16.7) (*n* = 8932)			72.7 (16.8) (*n* = 380)	70.5 (17.5) (*n* = 2350)			71.5 (18.7) (*n* = 192,463)	sig.	−0.14		
Role conflicts (high = negative)	52.1 (18.6) (*n* = 564)	51.9 (21.6) (*n* = 8918)			45.1 (19.6) (*n* = 380)	49.3 (20.6) (*n* = 2.347)	sig.	0.21	45.7 (23.3) (*n* = 192,044)	+	−0.27		
Quality of leadership (high = positive)	53.8 (22.7) (*n* = 543)	49.9 (25.3) (*n* = 8875)	sig.	−0.16	49.2 (22.9) (*n* = 369)	48.6 (23.6) (*n* = 2296)			51.4 (25.4) (*n* = 189,209)				
Social support (high = positive)	66.7 (17.0) (*n* = 564)	65.9 (19.8) (*n* = 8938)			64.2 (17.0) (*n* = 380)	64.4 (18.8) (*n* = 2334)			66.1 (21.1) (*n* = 192,147)				
Feedback (high = positive)	41.9 (21.0) (*n* = 564)	40.4 (22.2) (*n* = 8928)			41.0 (21.5) (*n* = 380)	40.1 (20.7) (*n* = 2330)			43.0 (22.5) (*n* = 191,336)				
Social relations (high = positive)	39.5 (28.7) (*n* = 564)	52.9 (24.7) (*n* = 8897)	–	0.54	46.7 (27.5) (*n* = 380)	50.4 (26.6) (*n* = 2324)			54.0 (28.5) (*n* = 190,298)	–	0.51	–	0.26
Sense of community (high = positive)	77.1 (15.0) (*n* = 564)	73.5 (16.3) (*n* = 8935)	sig.	−0.22	78.0 (14.3) (*n* = 380)	76.5 (15.3) (*n* = 2328)			76.2 (18.7) (*n* = 191,074)				
**Domain: Strain (effects, outcomes)**													
Job satisfaction (high = positive)	66.7 (10.6) (*n* = 564)	57.8 (16.7) (*n* = 8888)	+	−0.54	72.7 (12.6) (*n* = 380)	62.4 (16.8) (*n* = 2323)	+	−0.63	62.3 (16.9) (*n* = 190,431)	sig.	−0.26	+	−0.61

Significant differences in means of > = 5 are marked with a “+” (=study group value for WorkSafeMed is higher than COPSOQ database reference value) or by a “- “(study group value for WorkSafeMed is lower than COPSOQ database reference value); further differences not reaching the 5-point difference but significant with at least *p* < 0.01 indicated with a “sig.”

### WorkSafeMed nurses versus COPSOQ nurses

The comparison of the scales between WorkSafeMed nurses and COPSOQ nurses revealed a mixed picture. For three scales, we discovered statistically significant differences with medium to large effects. WorkSafeMed nurses indicated a higher (= better) level of *“job satisfaction”* (66.7 vs. 57.8). However, they also rated *“quantitative demands”* higher (68.4 vs. 61.9) and *“social relations”* lower (39.5 vs. 52.9) (i.e. worse) than the corresponding reference values for COPSOQ nurses. For five other scales, we found significantly better values for WorkSafeMed nurses, but with a rather small effect size: *“influence at work”* (41.5 vs. 37.3), *“possibilities for development”* (70.7 vs. 65.9), *“predictability”* (53.3 vs. 50.4), *“quality of leadership”* (53.8 vs. 49.9) and *“sense of community”* (77.1 vs. 73.5). Concerning *“work-privacy-conflict”*, the values were slightly higher (i.e. worse) for WorkSafeMed nurses (59.8 vs. 55.4) than for the reference group, representing only a small effect. For eight scales (*“control over working time”, “meaning of work”, “workplace commitment”, “role clarity”, “role conflicts”, “social support”, “feedback”, and “social relations”*), we found no statistically significant differences and values were in a similar range.

### WorkSafeMed nurses versus COPSOQ all occupations

The comparison with reference values for COPSOQ all occupations showed significantly poorer values with medium to large effects for the following scales: *“quantitative demands”* (68.4 vs. 56.3), *“work-privacy-conflict”* (59.8 vs. 42.7), *“control over working time”* (51.0 vs. 61.5), *“workplace commitment”* (49.8 vs. 58.3) and, *“social relations”* (39.5 vs. 54.0). WorkSafeMed nurses also indicated more *“role conflicts”* (52.1 vs. 45.7). This difference was significant but represented a small effect. In further comparisons, we found significantly better values for WorkSafeMed nurses with small to medium effects for the scales *“possibilities for development”* (70.7 vs. 61.9), *“meaning of work”* (83.0 vs. 74.6), *“role clarity”* (74.2 vs. 71.5), and *“job satisfaction”* (66.7 vs. 62.3). For seven scales (*“influence at work”, “predictability”, “quality of leadership”, “social support”, “feedback”, “social relations”, and “sense of community”*), we found no statistically significantly differences between the two groups.

### WorkSafeMed physicians versus COPSOQ physicians

The comparison of WorkSafeMed physicians and COPSOQ physicians revealed several statistically significant differences. WorkSafeMed physicians stated slightly higher *“quantitative demands”* than the reference group (73.9 vs. 70.1). This difference represented only a small effect. For four other scales, we found better values for WorkSafeMed physicians with small to medium effects. WorkSafeMed physicians rated *“meaning of work”* higher (85.9 vs. 81.8) and *“role conflicts”* lower (45.1 vs. 49.3) than the corresponding reference values. *“Possibilities for development”* (80.1 vs. 75.0) and *“workplace commitment”* (63.0 vs. 53.6) were also assessed more positively by WorkSafeMed physicians. Concerning *“job satisfaction”*, we found a significant difference between the two samples. WorkSafeMed physicians indicated higher *“job satisfaction”* than the COPSOQ physicians (72.7 vs. 62.4). This difference represented a large effect. For the remaining 10 scales (*“work-privacy-conflict”, “influence at work”, “control over working time”, “predictability”, “role clarity”, “quality of leadership”, “social support”, “feedback”, “social relations”, and “sense of community”*)*,* no statistically significant differences between the two groups were detectable.

### WorkSafeMed physicians versus COPSOQ all occupations

The comparison between WorkSafeMed physicians and reference values from COPSOQ all occupations was similar to the comparison of WorkSafeMed nurses: We found significantly poorer values for *“control over working time”* (52.6 vs. 61.5) and *“social relations”* (46.7 vs. 54.0). This difference represented a small to medium effect. We identified significantly higher *“quantitative demands”* (73.9 vs. 56.3) and a higher *“work-privacy-conflict”* (68.8 vs. 42.7) for WorkSafeMed physicians with a rather large effect size. In further comparisons, we found significantly better values for *“workplace commitment”* (63.0 vs. 58.3), *“possibilities for development”* (80.1 vs. 61.9), *“meaning of work”* (85.9 vs. 74.6) and *“job satisfaction”* (72.7 vs. 62.3). The differences for *“possibilities for development”*, *“meaning of work”* and *“job satisfaction”* represented a large effect, while the difference for *“workplace commitment”* showed only a small effect size. For nine scales (*“influence at work”, “predictability”, “role clarity”, “role conflicts”, “quality of leadership”, “social support”, “feedback”, “social relations”, and “sense of community”*), we identified no statistically significantly differences between the two groups. Values were within a similar range.

## Discussion

In this study, we applied a multistep approach to convert COPSOQ scales from the WorkSafeMed study (G-COPSOQ II) to the COPSOQ scales from the current German COPSOQ database (G-COPSOQ III). We then compared the converted WorkSafeMed scales with corresponding reference data from the German COPSOQ database.

### Preliminary work

The explorative statistical analysis included different procedures to examine original and newly converted WorkSafeMed scales and was performed after a comprehensive comparison at the content level. A newly published study on COPSOQ III, conducted in Canada, Spain, France, Germany, Sweden, and Turkey, also highlighted the differences in content between the international version of COPSOQ II and COPSOQ III [3]. This content-based explorative approach was, in our opinion, suitable for finding relevant differences between the original scales of the questionnaire used within the WorkSafeMed study and the converted WorkSafeMed scales.

The performed reliability analysis resulted for most of the original and converted WorkSafeMed scales in satisfying Cronbach’s alpha values above .70. Unfortunately, the results of the German validation study for G-COPSOQ III have not yet been published. But compared with respective values from the validation studies of G-COPSOQ I and the international COPSOQ III [3, 19], we identified in most cases similar values. In some cases, we had lower Cronbach’s alpha values in our sample compared to results from other validation studies [3, 19]. We detected lower values especially for four original WorkSafeMed-scales (*“social relations”, “control over working time”, “predictability”, and “feedback”*) and also for four converted WorkSafeMed scales (*“quantitative demands”, “control over working time”, “predictability”, and “feedback”*). A possible explanation for these low values is certainly that Cronbach alpha is influenced by the number of items [28]. The affected scales have on average only two items. Scales that contain more items usually have higher Cronbach’s alpha values [28]. On top, specific answering patterns of our sample of nurses and physicians have to be considered.

Based on our sample, we found clear differences for the original and converted WorkSafeMed scales *“control over working time”* and *“social relations”*.

The difference in scale composition for the scale *“control over working time”* may explain the higher mean value for the converted scale than for the original scale (51.8 vs. 40.2), and the high measures for effect size (d_Cohen_ = 0.56) and nomenclature (> 10 points). The original scale *“control over working time”* was reduced by the following two single items (“Can you leave your work to have a chat with a colleague?” / “If you have some private business, is it possible for you to leave your place of work for half an hour without special permission?”). We detected for the first item a ceiling effect of more than 80%. For the other item, we could not find any floor or ceiling effects. This effect is probably explained by the special work environment of nurses and physicians in the present case. Nurses and physicians can neither “leave the place of work to have a chat” nor can they “leave the place of work for half an hour without special permission” due to the special work circumstances. Thus, the original scale did not fit well for the hospital workplace. The sample’s agreement and the variance within the two items of the converted scale were higher than with the other two items of the original scale. The converted scale included only single items that are relevant for work in hospitals.

For the scale *“social relations”*, the lower mean derived applying the converted scale (i.e. single item) may capture the situation of employees in hospitals better than the original scale (41.8 vs. 55.6). Additionally, the effect size (d_Cohen_ = − 0.55) and nomenclature (> 10 points) of this difference are high, indicating a clear difference between original and converted scales. The converted scale *“social relations”* was reduced by one single item (“Do you work separate from your colleagues?”). This item also was not formulated appropriately for hospital work. The work in hospitals is characterized by a frequent turnover of patients, some processes take place in a team, and some tasks are carried out by persons alone. Therefore, the converted scale included only one single item “Is it possible for you to talk to your colleagues while you are working?” which may well depict this aspect of work in the hospital environment.

As for the other scales, the differences between original and converted WorkSafeMed scales were not relevant; a comparison of our converted data with data from the current German COPSOQ database, as well as with current studies applying the new COPSOQ version was possible.

### Comparison of converted WorkSafeMed scales with reference data

After reconstructing the scales, we compared 16 converted scales from the WorkSafeMed study with corresponding reference data from the current German COPSOQ database.

The values for WorkSafeMed and COPSOQ nurses showed a rather typical appearance of the nursing profession with high values for *“quantitative demands”* and *“work-privacy-conflict”*, but also positive results for *“meaning of work”* and *“sense of community”*. Other studies also indicated high levels of job stress and work burden among German nurses and physicians [29, 30]. The comparison between our sample and the database revealed better values for WorkSafeMed nurses for the scales *“job satisfaction”, “influence at work”, “possibilities for development”, “predictability”, “quality of leadership”*, and *“sense of community”*. We identified worse values for the scales *“quantitative demands”, “social relations”*, and *“work privacy conflict”*. These differences are maybe on the one hand due to the different work settings (university hospital versus general hospital). On the other hand, the WorkSafeMed nurses covered a smaller sample and cannot be considered as representative compared to other nurses in general hospitals. In summary, the comparative results should be interpreted with caution as all effect sizes were only small except for the scales *“job satisfaction”, “quantitative demands”*, and *“social relations”* with medium effect sizes.

The values for WorkSafeMed and COPSOQ physicians also represented well-known findings for this medical profession. Physicians in hospitals had to struggle with high *“quantitative demands”,* and a high *“work-privacy-conflict”*. A recent study showed that high perceived psychosocial stress and extended working time were associated with a higher rate of physicians’ intention to leave direct patient care [31]. But physicians also reported positively about *“possibilities for development”* and *“meaning of work”.* The comparison between our sample and the database demonstrated in some scales (*“meaning of work”, “role conflicts”, “possibilities for development”, “workplace commitment”,* and *“job satisfaction”*) better values for the WorkSafeMed physicians. Only the scale *“quantitative demands”* was rated worse by the WorkSafeMed physicians. Some differences may be also linked to the different workplaces. Physicians at university hospitals are often simultaneously involved in patient care, teaching, and research, and therefore perceive a high level of quantitative demands. However, likewise, the sample of WorkSafeMed physicians cannot be regarded as representative for other physicians at general hospitals, so also the identified differences should be interpreted with caution.

Compared with COPSOQ data on all occupations, we identified higher *“quantitative demands”* and lower values for *“social relations”* in the WorkSafeMed sample. WorkSafeMed nurses and physicians had also to struggle with a higher *“work-privacy-conflict”* compared to other professions. The difficulty of combining requirements from working and private life is also reported in other studies using a comparable scale for work-privacy-conflict: the work-family-conflict scale by Netemeyer [32, 33]. As part of his COPSOQ validation study in 2004, Nübling et al. used a modified version of the work-family-conflict scale by Netemeyer and thus replaced the term family with the term privacy in the name of the scale [9]. In a recent study, Mache et al. examined working conditions and work-family-conflict in the medical profession in 15 hospitals in Germany by means of G-COPSOQ II [33]. They found similarly high levels of work-family conflict (mean = 76) and quantitative demands (mean = 75) among German hospital physicians [33]. In 2005, Fuß et al. surveyed physicians regarding their perceived work-family conflict and their working conditions in two university hospitals in Germany with G-COPSOQ I, too [32]. They also discovered high levels of work-family-conflict (mean = 74) and higher quantitative demands (mean = 73) compared to the general German working population as depicted in the then-current COPSOQ database [32]. On the basis of our comparison undertaken with reference data from the current German COPSOQ database, as well as with regard to the correlation of perceived psychosocial working conditions in hospitals and quality of patient care [18, 34–39], it is all in all crucial to reduce high quantitative demands and high work-privacy-conflicts of physicians and nurses in Germany. Therefore, measures at the legislative level in Germany are necessary to further-reduce high quantitative demands for nurses and physicians [40]. Based on the high correlation of documented work overtime and perceived high quantitative demands, as well as high work-privacy-conflict in physicians [18], comprehensive measures should be implemented leading to an effective adherence to (daily and weekly) maximum working hours like e.g. new shift models [41–43].

Another interesting finding in the WorkSafeMed sample was a surprisingly high *“job satisfaction”* despite high *“quantitative demands”*. One possible explanation for this result can be found in the work setting (university hospital versus non-university hospital). University hospitals offer to physicians and nurses a variety of learning opportunities due to interesting and complex treatment cases. Further training and qualification opportunities at a university hospital can also contribute to high job satisfaction. However, the results from a standard assessment of job satisfaction with classical global ratings should generally be considered with caution. A recent study by Hiemisch et al. considered the discrepancy between challenging working conditions and subjective job satisfaction [44]. In their study, the authors conducted an assessment of job satisfaction using classical global ratings and additionally included the measurement of qualitative job satisfaction based on the cognitive-emotional concept of the “Schweizer Modell” [44]. According to the global rating, they found a high level of job satisfaction among the medical, nursing, and administrative/technical staff [44]. In contrast, however, the additional analysis showed that only 1 in 4 employees was actually satisfied with his or her job [44]. The authors concluded that the assessment with classical global ratings was not appropriate, because it showed only responses of resignatively satisfied employees and missed perceptions of unsatisfied employees [44].

In accordance to other studies [45–47], we found low values for *“control over working time”* and high values for *“possibilities for development”* and *“meaning of work”* among the WorkSafeMed sample. In our opinion, these results are typical for the two professions and for the work in university hospitals. The work is characterized by mandatory regulations and standards for both professions. Therefore, it can be assumed that physicians and nurses perceive that they actually have little control over their own working time. The high demands in this specific environment lead - especially in the setting of university hospitals investigated in the WorkSafeMed study - to high values for *“possibilities for development”* and for *“meaning of work”*. According to Leape and colleagues, it is crucial for healthcare organisations to create a working environment where employees find meaning in their work [48]. This can be encouraged by the following measures: every employee is treated with respect, has the possibility (by education, training, encouragement) to make an essential contribution at work that gives meaning to their life, and feel valued for what they do [48].

### Strength and limitations

The WorkSafeMed study was not designed to compare both COPSOQ versions. However, in our opinion, the explorative approach chosen to convert COPSOQ scales used in the WorkSafeMed study to G-COPSOQ III and to compare both versions statistically was appropriate. In addition, this may present a good possibility for other COPSOQ studies that used G-COPSOQ II to compare their results with more current data. In general, our explorative approach can be applied in other studies to compare findings gathered with different versions of a questionnaire used e.g. in different research projects over time. As not only the COPSOQ but also other questionnaires may be developed further it seems crucial to report all respective details of the questionnaire (e.g. version, year) used in a research project to enable the correct comparison with results from other studies.

Our approach also made it possible to look more critically at single items of the original COPSOQ questionnaire (G-COPSOQ II) for the hospital sector. The comparison with corresponding reference data from the current COPSOQ database proved to be valuable, and possible implications for improved psychosocial working conditions could be identified, e.g. reduction of high quantitative demands and high work-privacy-conflicts of physicians and nurses at university hospitals in Germany.

We can also address some limitations in our study. We developed an explorative approach to compare and convert scales. Unfortunately, there are currently few studies that describe such a scale adjustment. Therefore, we cannot refer to any validated methodology for our explorative approach. The WorkSafeMed study included a cross sectional design with subjective judgements of self-reported data from nurses and physicians. The reference data comprised nurses and physicians from both general hospitals and university hospitals often taking part in the survey as one step of the psychosocial risk assessment. The different work setting, particular in general hospitals, and the different embedding of the survey may explain some of the identified differences. Thus, the different results must be considered with caution. Also, the data from the WorkSafeMed study comprised a smaller sample and was based on only two university hospitals in Germany. We obtained in the WorkSafeMed sample a response rate of 39.6%. This response rate is quite high for surveys with nurses and physicians in the German hospital sector. Unfortunately, it was not possible to conduct a non-responder analysis to identify potential differences and to assess whether the WorkSafeMed sample can be considered representative for nurses and physicians in German university hospitals. Thus, we cannot completely rule out a possible response bias and that the results may be representative for neither other medical professions in university hospitals nor for all hospitals in Germany. Furthermore, the survey data used (WorkSafeMed and reference data from the COPSOQ database) originated from surveys conducted at different times. Therefore, also time trends might account for some of the identified differences. The high mean values for the scale job satisfaction together with scales illustrating the high psychosocial strain suggest that resignatively satisfied employees also took part in the survey. In future studies, a more differentiated measurement of job satisfaction would help to detect potentially dissatisfied employees.

## Conclusions

In this study, we performed an explorative approach for the conversion of WorkSafeMed scales (G-COPSOQ II) for hospital nurses and physicians to the current version of the German COPSOQ questionnaire (G-COPSOQ III). In our opinion, the conversion of WorkSafeMed scales was possible and appropriate and thus allowed a comparison between three reference values in the current German COPSOQ database. The comparison with reference values revealed some implications for the improvement of psychosocial working conditions of nurses and physicians which should be considered in university hospitals in Germany. In all studies, enough details on the questionnaires used for data assessment (i.e. version, year) should be published to enable comparative analyses.

## Data Availability

The reference data are from the COPSOQ database of FFAW GmbH. Due to data security aspects, data from the WorkSafeMed study will not be made available to the public domain. However, data will be used by students of both project partners for their theses. Data will be stored in accordance with national and regional data security standards.

## Abbreviations

COPSOQ: Copenhagen Psychosocial Questionnaire
G-COPSOQ I: German standard version based on Copenhagen Psychosocial Questionnaire I
G-COPSOQ II: German standard version based on Copenhagen Psychosocial Questionnaire I and II
G-COPSOQ III: German standard version based on Copenhagen Psychosocial Questionnaire III
WorkSafeMed: Working conditions, safety culture, and patient safety in hospitals – what predicts the safety of the medication process

## References

[1] Kristensen TS, Hannerz H, Høgh A, Borg V (2005). The Copenhagen psychosocial questionnaire—a tool for the assessment and improvement of the psychosocial work environment. Scand J Work Environ Health.

[2] Pejtersen JH, Kristensen TS, Borg V, Bjorner JB (2010). The second version of the Copenhagen psychosocial questionnaire. Scand J Public Health..

[3] Burr H, Berthelsen H, Moncada S, Nübling M, Dupret E, Demiral Y (2019). The third version of the Copenhagen psychosocial questionnaire. Saf Health Work.

[4] Berthelsen H, Westerlund H, Bergström G, Burr H. Validation of the Copenhagen psychosocial questionnaire version III and establishment of benchmarks for psychosocial risk Management in Sweden. Int J Environ Res Public Health. 2020. 10.3390/ijerph17093179. 10.3390/ijerph17093179 PMC7246423 32370228

[5] Şahan C, Baydur H, Demiral Y (2019). A novel version of Copenhagen psychosocial Questionnaire-3: Turkish validation study. Arch Environ Occup Health.

[6] Berthelsen H, Hakanen JJ, Westerlund H (2018). Copenhagen psychosocial questionnaire - a validation study using the job demand-resources model. PLoS One.

[7] Rosário S, Azevedo LF, Fonseca JA, Nienhaus A, Nübling M, da Costa JT (2017). The Portuguese long version of the Copenhagen psychosocial questionnaire II (COPSOQ II) - a validation study. J Occup Med Toxicol.

[8] Moncada S, Utzet M, Molinero E, Llorens C, Moreno N, Galtés A, Navarro A (2014). The Copenhagen psychosocial questionnaire II (COPSOQ II) in Spain--a tool for psychosocial risk assessment at the workplace. Am J Ind Med.

[9] Nübling M, Stößel U, Hasselhorn HM, Michaelis M, Hofmann F (2005). Methoden zur Erfassung psychischer Belastungen: Erprobung eines Messinstrumentes (COPSOQ) [methods for the assessment of mental work load – testing of a measuring procedure (COPSOQ)].

[10] Nübling M, Hasselhorn HM (2010). The Copenhagen psychosocial questionnaire in Germany: from the validation of the instrument to the formation of a job-specific database of psychosocial factors at work. Scand J Public Health.

[11] Nübling M, Vomstein M, Haug A, Nolle I, Lindner A, Lincke HJ (2018). COPSOQ 3: Internationale Weiterentwicklung und deutsche Standardversion [COPSOQ 3: international development and German standard version]. Psychother Psych Med.

[12] COPSOQ.de (2020). Scientific research and risk assessment with the Copenhagen Psychosocial Questionnaire (COPSOQ) in Germany.

[13] GDA (2014). Recommendations of the institutions of the Joint German Occupational Safety and HealthStrategy (GDA) for implementing psychosocial risk assessment.

[14] Kiss P, de Meester M, Kruse A, Chavée B, Braeckman L. Comparison between the first and second versions of the Copenhagen Psychosocial Questionnaire: psychosocial risk factors for a high need for recovery after work. Int Arch Occup Environ Health. 2013. 10.1007/s00420-012-0741-0. 10.1007/s00420-012-0741-0 22302351

[15] Wagner A, Hammer A, Manser T, Martus P, Sturm H, Rieger MA. Do occupational and patient safety culture in hospitals share predictors in the Field of psychosocial working conditions? Findings from a cross-sectional study in German University hospitals. Int J Environ Res Public Health. 2018. 10.3390/ijerph15102131. 10.3390/ijerph15102131 PMC6210136 30262790

[16] Wagner A, Rieger MA, Manser T, Sturm H, Hardt J, Martus P (2019). Healthcare professionals’ perspectives on working conditions, leadership, and safety climate: a cross-sectional study. BMC Health Serv Res.

[17] Hammer A, Wagner A, Rieger MA, Manser T (2019). Assessing the quality of medication documentation: development and feasibility of the MediDocQ instrument for retrospective chart review in the hospital setting. BMJ Open.

[18] Sturm H, Rieger MA, Martus P, Ueding E, Wagner A, Holderried M, Maschmann J (2019). Do perceived working conditions and patient safety culture correlate with objective workload and patient outcomes: A cross-sectional explorative study from a German university hospital. PLoS One.

[19] Nübling M, Stößel U, Hasselhorn HM, Michaelis M, Hofmann F (2006). Measuring psychological stress and strain at work: evaluation of the COPSOQ questionnaire in Germany. GMS Psycho Soc Med.

[20] Schafer JLGJ (2002). Missing data: our view of thes state of the art. Psychol Methods.

[21] Wirtz M (2004). Uber das problem fehlender Werte: Wie der Einfluss fehlender Informationen auf Analyseergebnisse entdeckt und reduziert werden kann [on the problem of missing data: how to identify and reduce the impact of missing data on findings of data analysis]. Rehabilitation (Stuttg).

[22] Field A (2013). Discovering statistics using IBM SPSS statistics.

[23] Kline RB (2005). Principles and practice of structural equation modeling.

[24] Hemmerich W (2019). Rechner zur Adjustierung des α-Niveaus: StatistikGuru [calculator for adjusting the α level].

[25] Bühner MZM (2009). Statistik für Psychologen und Sozialwissenschaftler [statistics for psychologists and social scientists].

[26] Kersten M, Kozak A, Wendeler D, Paderow L, Nübling M, Nienhaus A (2014). Psychological stress and strain on employees in dialysis facilities: a cross-sectional study with the Copenhagen psychosocial questionnaire. J Occup Med Toxicol.

[27] Nübling M, Vomstein M, Schmidt SG, Gregersen S, Dulon M (2010). Psychosocial work load and stress in the geriatric care. BMC Public Health.

[28] Cortina JM (1993). What is coefficient alpha? An examination of theory and applications. J Appl Psychol.

[29] Büssing A, Falkenberg Z, Schoppe C, Recchia DR, Poier D (2017). Work stress associated cool down reactions among nurses and hospital physicians and their relation to burnout symptoms. BMC Health Serv Res.

[30] Sehlen S, Vordermark D, Schäfer C, Herschbach P, Bayerl A, Pigorsch S (2009). Job stress and job satisfaction of physicians, radiographers, nurses and physicists working in radiotherapy: a multicenter analysis by the DEGRO quality of life work group. Radiat Oncol.

[31] Degen C, Li J, Angerer P (2015). Physicians’ intention to leave direct patient care: an integrative review. Hum Resour Health.

[32] Fuss I, Nübling M, Hasselhorn H-M, Schwappach D, Rieger MA (2008). Working conditions and work-family conflict in German hospital physicians: psychosocial and organisational predictors and consequences. BMC Public Health.

[33] Mache S, Bernburg M, Vitzthum K, Groneberg DA, Klapp BF, Danzer G (2015). Managing work-family conflict in the medical profession: working conditions and individual resources as related factors. BMJ Open.

[34] Aiken LH, Clarke SP, Sloane DM, Sochalski J, Silber JH (2002). Hospital nurse staffing and patient mortality, nurse burnout, and job dissatisfaction. JAMA..

[35] Aiken LH, Sloane DM, Bruyneel L, van den Heede K, Sermeus W (2013). Nurses’ reports of working conditions and hospital quality of care in 12 countries in Europe. Int J Nurs Stud.

[36] Aiken LH, Sloane DM, Bruyneel L, van den Heede K, Griffiths P, Busse R (2014). Nurse staffing and education and hospital mortality in nine European countries: a retrospective observational study. Lancet.

[37] McHugh M, Rochman M, Sloane D, Berg R, Mancini M, Nadkarni V, Merchant R, Aiken LH (2016). Better nurse staffing and nurse work environments associated with increased survival of in-hospital cardiac arrest patients. Med Care.

[38] Weissman J, Rothschild J, Bendavid E, Sprivulis P, Cook E, Evans R, Kaganova Y, Bender M, David-Kasdan J, Haug P, Lloyd J, Selbovitz L, Murff H, Bates D (2007). Hospital workload and adverse events. Med Care.

[39] Friese CR, Lake ET, Aiken LH, Silber JH, Sochalski J (2008). Hospital nurse practice environments and outcomes for surgical oncology patients. Health Serv Res.

[40] Sozialgesetzbuch (SGB). Elftes Buch (XI) – Soziale Pflegeversicherung – (SGB XI) [Book eleven (XI) - Long-term care social insurance] (Artikel 1 des Gesetzes vom 26. Mai 1994 – BGBl. I S. 1014, ber. S. 2797).

[41] Maschmann J, Holderried M, Blumenstock G, Bamberg M, Rieger MA, Tatagiba M, Roser F (2012). Acta Neurochir (Wien).

[42] Maschmann J, Holderried M, Blumenstock G, Rieger MA, Bamberg M, Rosenberger P, Wagner T (2012). Neues Dienstzeitenmodell für Ärzte in der Anästhesie : Eine analyse 3 Jahre nach Implementierung [a new working shift model for anesthesiologists: an analysis 3 years after implementation]. Anaesthesist..

[43] Maschmann J, Holderried M, Blumenstock G, Bamberg M, Rieger M, Wallwiener D, Brucker S (2013). Impact of new shift models for doctors working at a German University hospital for Gynaecology and obstetrics four years after implementation. Can they meet the European working time directive without increasing costs?. Geburtsh Frauenheilk.

[44] Hiemisch A, Stöbel-Richter Y, Grande G, Brähler E, Kiess W (2019). Sind wir wirklich so glücklich, wie wir es glauben? Eine kritische Untersuchung der Arbeitszufriedenheit an einer Universitätskinderklinik [are we really as happy as we think we are? A critical examination of work satisfaction in a university pediatric hospital]. Gesundheitswesen..

[45] Schmidt S, Bartholomeyczik S, Dieterle WE, Wittich A, Donath E, Rieger MA (2008). Arbeitsbedingungen für die Pflege in Krankenhäusern als Herausforderung. Eine Sekundäranalyse der Basiserhebung im Forschungsprojekt “Arbeitsbedingungen im Krankenhaus” (ArbiK) [working conditions of nurses in hospitals as a challenge. A secondary analysis of the baseline data collection of the ArbiK-project]. Pflege & Gesellschaft.

[46] Bernburg M, Vitzthum K, Groneberg DA, Mache S (2016). Physicians’ occupational stress, depressive symptoms and work ability in relation to their working environment: a cross-sectional study of differences among medical residents with various specialties working in German hospitals. BMJ Open.

[47] Mache S, Vitzthum K, Nienhaus A, Klapp BF, Groneberg DA (2009). Physicians’ working conditions and job satisfaction: does hospital ownership in Germany make a difference?. BMC Health Serv Res.

[48] Leape L, Berwick D, Clancy C, Conway J, Gluck P, Guest J (2009). Transforming healthcare: a safety imperative. Qual Saf Health Care.

